# Computational Research of the Efficiency of Using a Three-Layer Panel Made of Highly Porous Polystyrene Concrete

**DOI:** 10.3390/ma17164133

**Published:** 2024-08-21

**Authors:** Galiya Rakhimova, Nurlan Zhangabay, Tatyana Samoilova, Murat Rakhimov, Pyotr Kropachev, Victor Stanevich, Murat Karacasu, Ulzhan Ibraimova

**Affiliations:** 1 Department of Construction Materials and Technologies, Abylkas Saginov Karaganda Technical University, Karaganda 100000, Kazakhstan; galinrah@mail.ru (G.R.); tanya.fedulova.18@mail.ru (T.S.); kropachev-54@mail.ru (P.K.); 2 Department of Architecture and Urban Planning, M. Auezov South Kazakhstan University, Shymkent 16000, Kazakhstan; ibraimova_uljan@mail.ru; 3 Department of Architecture and Construction, Toraighyrov University, Pavlodar 140000, Kazakhstan; 4 Department of Architecture and Civil engineering, Eskisehir Technical University, 26040 Eskisehir, Turkey; muratk@ogu.edu.tr

**Keywords:** three-layer panel, polystyrene concrete, temperature field, thermal resistivity, humidity conditions, permeability to air, modeling

## Abstract

This paper presents linking computational research of the multilayer structure of the cladding of a three-layer panel made of highly porous polystyrene concrete developed using a new technology in comparison with traditional ones. The calculation of the thermal efficiency of the exterior fence was carried out in three stages, where the thermal regime was calculated from the values of temperature fields in the ELCUT 6.6 system, and the humidity and air modes were determined by the analytical method in the Maple system. The territory of central Kazakhstan (Karaganda) was selected as the research region, where the research showed that equating the thickness by the values of the actual and required heat transfer resistances of traditional multilayer structures to the developed one, the thickness of traditional structures increases from 3.09% to 27.83%. Moisture accumulation relative to the developed one occurs in all the studied structures. Thus, if in some cases of traditional structures moisture is collected by 2.61% and 9.48% less, in others moisture is collected by 27.94% and 119% more. However, the value of evaporated moisture during the drying period showed that all the moisture will evaporate during the specified period. Thus, all the structures meet the conditions for the inadmissibility of moisture for the annual period and the period of moisture accumulation. Moreover, the values of the actual and required permeabilities to air satisfy the condition, which affected the values of the temperature fields taking into account air filtration; the developed structure showed a positive effect for this value, and in traditional structures, the value of $\tau_{int}$ decreased to 1.35 °C depending on the option. The analytical results of the thermal inertia values of the developed and traditional multilayer structures showed that the developed structure exceeds traditional ones by up to 30.04% depending on the option, which is positive in the cold period. It was also found that the market prices of all traditional structures exceed the developed one by 1.2–2.5 times, depending on the design, which also emphasizes the positive aspects of the new design. Thus, the findings of this research will positively complement the catalog of products of external multilayer cladding structures made of effective materials and can be used by research communities and design organizations in the design of residential buildings.

## 1. Introduction

Today, the housing and utilities sector is one of the most energy-intensive industries of the Republic of Kazakhstan, consuming almost 65% of the country’s fuel and energy resources [1], where 70% of the total housing sector is made up of apartment buildings, the number of which has exceeded 18 thousand buildings [2]. In this regard, the development of new measures aimed at improving the rules for accounting and monitoring energy consumption and maximum energy losses is of particular importance, where one of the priority areas of energy saving in the housing and utilities sector is increasing the energy efficiency of the housing stock [3,4,5,6]. Most buildings in the Republic have external cladding structures with inefficient or economically inexpedient indicators that do not correspond to modern trends in the development of enclosures in the context of international experience, which is fraught with excessive consumption of thermal energy [7,8].

Currently, there is a lot of development and research being conducted on outdoor fences on an international scale and on the scale of the Republic of Kazakhstan. The importance of such research around the world is determined by the optimal design of outdoor fences, where engineers and scientists must take into account issues such as economically efficient and energy-efficient structures of outdoor fences, which is extremely difficult. Research in this area on an international scale will help to understand more clearly which of the designs is most acceptable in certain situations. Thus, domestic scientists in [9,10,11] studied the outer shell option using heat-accumulating material for free and forced convection. The efficiency of using this design was up to 44%; however, in multi-apartment residential buildings, the use of this enclosure is problematic, and to achieve the specified effect, this design is applicable only for southern Kazakhstan. In studies [12,13,14,15], the authors analyzed the efficiency of curtain facade systems with air gaps in comparison with the traditional one. However, as is known, curtain facade systems with ventilated layers are much more expensive than conventional wet facades, the use of which will affect the pricing of the building [16].

On an international scale, the problem of enclosures also has a significant emphasis, where various enclosure designs and methods for achieving them are studied in order to create an effective comfortable environment for human habitation. Thus, in [17], the authors studied the issue of optimizing traditional external walls, taking into account the orientation of the building to achieve a comfortable environment, where the effect was up to 6.59%. With that, the use of lightweight external walls was studied in [18], but this type of construction is not acceptable for apartment buildings. In the studies [19,20,21], the authors reviewed the possibility of using vacuum insulation panels in outer shells, where they reflected on the disadvantages and advantages of these panels; however, this type of thermal insulation in panels has not yet become widespread, which calls into question the widespread use of this panel in outer shells of apartment buildings [22,23,24].

The conducted review of research indicates the diversity of existing multilayer outer shells used in various climatic conditions. However, the review showed that the research conducted in this direction is insufficient, since there are no comparative studies in the field of thermophysical and economic indicators of external fences in the conditions of the Republic of Kazakhstan (Karaganda), which is a gap that requires additional research in this direction. In this regard, our research is aimed at a theoretical analysis of the thermal and physical and economic indicators of a three-layer panel made of highly porous polystyrene concrete [25,26] developed using a new technology in the climatic conditions of Karaganda, in comparison with existing traditional outer shells. The research is relevant, and its scientific novelty lies in obtaining energy-efficient outdoor fences.

## 2. Materials and Methods

### 2.1. Studied Options of Multilayer Structures of Outer Shells

**Figure 1:**
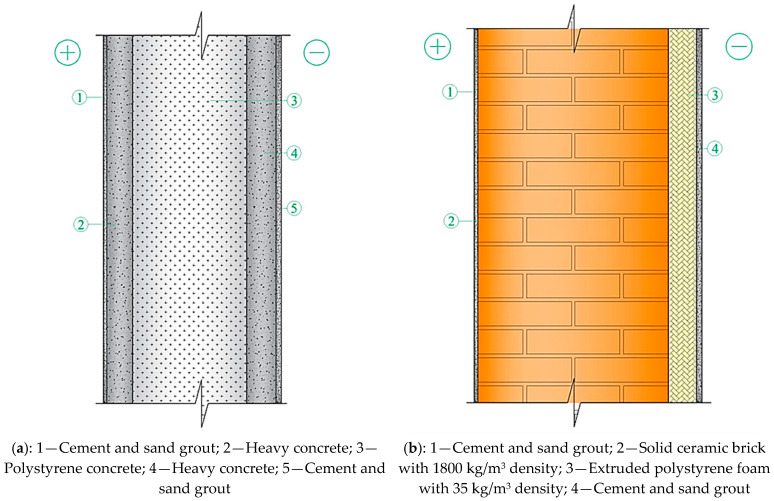

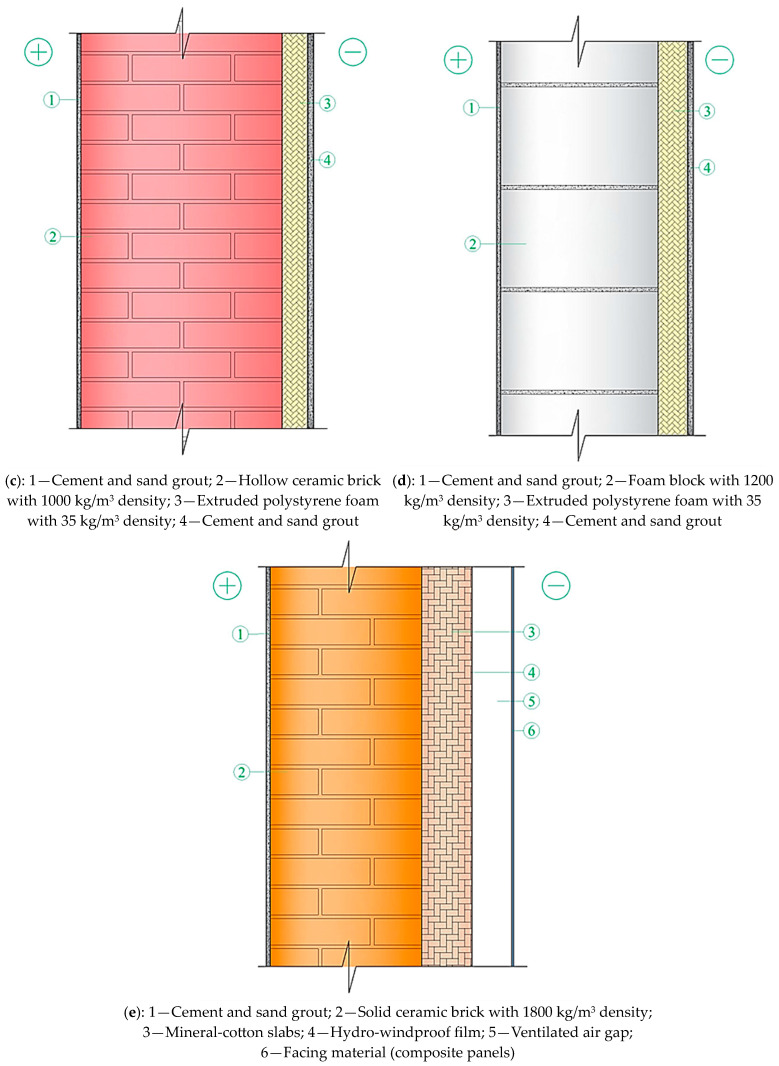
Options of multilayer structures of outer shells. An explanation of the numbering of the layers is given in Table 1, Table 2, Table 3, Table 4 and Table 5: (**a**)—three-layer panel made of highly porous polystyrene concrete (option 1); (**b**)—traditional enclosure made of solid ceramic brick (option 2); (**c**)—traditional enclosure made of hollow ceramic brick (option 3); (**d**)—traditional enclosure made of foam block (option 4); (**e**)—outer shell with a ventilated layer (option 5).

Our research examined five types of multilayer structures of outer shells, where a three-layer panel made of highly porous polystyrene concrete, obtained using a new technology [25] (Figure 1a), was proposed as a new design in comparison with existing structures of outer shells (Figure 1a,c–e).

The main thermal engineering characteristics of the studied multilayer structures of outer shells are presented in Table 1, Table 2, Table 3, Table 4 and Table 5 [27].

### 2.2. Outer Shells’ Thermal Efficiency Design Procedure

**Figure 2:**
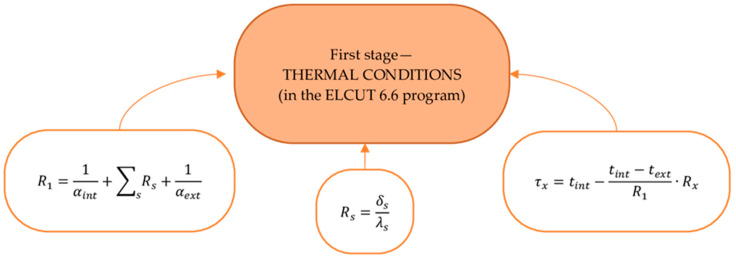
Algorithm for calculating the thermal conditions of the multilayer structures of the outer shells [30].

**Figure 3:**
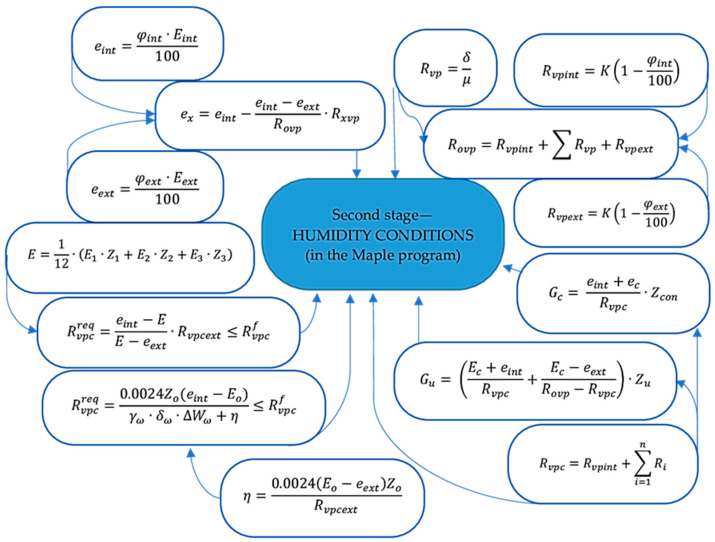
Algorithm for calculating the humidity conditions of the multilayer structures of the outer shells [25].

**Figure 4:**
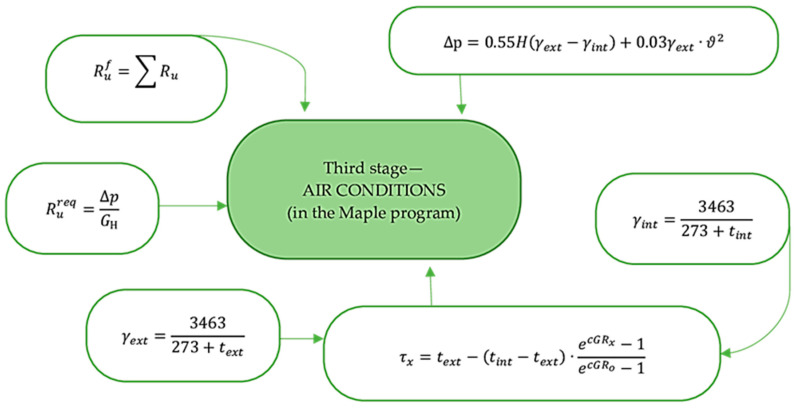
Algorithm for calculating the air conditions of the multilayer structures of the outer shells [25].

The thermal efficiency design procedure for the outer shells was carried out in three stages. Figure 2, Figure 3 and Figure 4 show the design procedure for all stages, which consist of thermal (Figure 2), humidity (Figure 3), and air conditions (Figure 4), carried out according to [27,28,29,30,31,32].

### 2.3. Climatic and Internal Boundary Conditions of the Region

The research examined a region located in the central part of the Republic of Kazakhstan. The main climatic indicators were adopted according to the standard [33,34] and are presented in Table 6.

## 3. Results and Discussion

### 3.1. Determination of the Actual Heat Transfer Resistance of the Multilayer Structures of the Outer Shells

**Figure 5:**
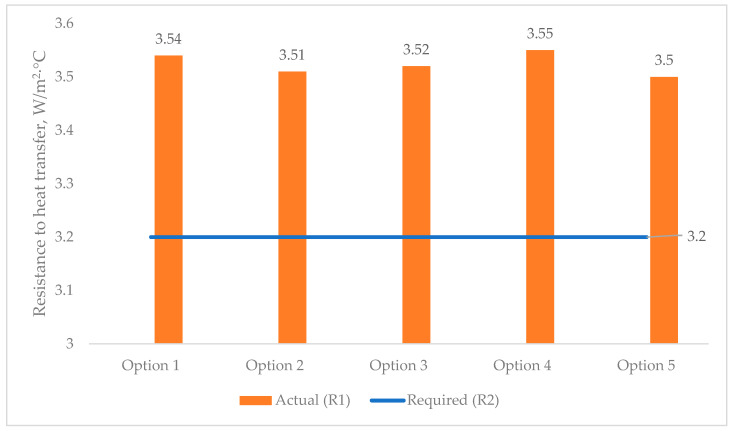
Values of actual (R1) and required (R2) heat transfer resistances of the multilayer structures of the outer shells: option 1—three-layer panel made of highly porous polystyrene concrete; option 2—traditional enclosure made of solid ceramic brick; option 3—traditional enclosure made of hollow ceramic brick; option 4—traditional enclosure made of foam block; option 5—outer shell with a ventilated layer.

Figure 5 shows the analytical results of the actual heat transfer resistances of all the multilayer structures of the outer shells (Figure 1) in comparison with the required one (Table 6), obtained according to [27,34].

### 3.2. Research of Temperature Distribution at the Boundaries of The Multilayer Structures of the Outer Shells

**Figure 6:**
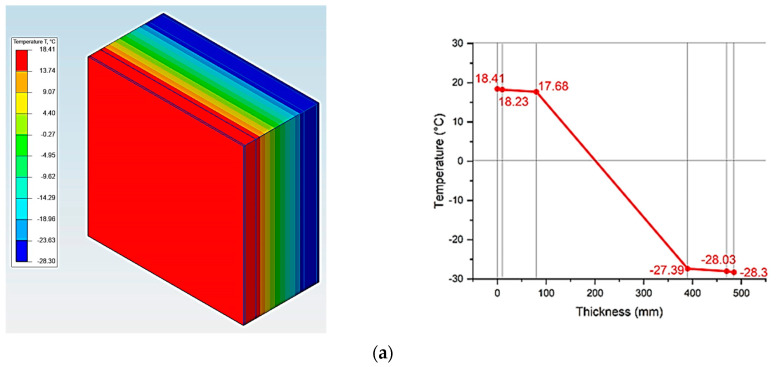

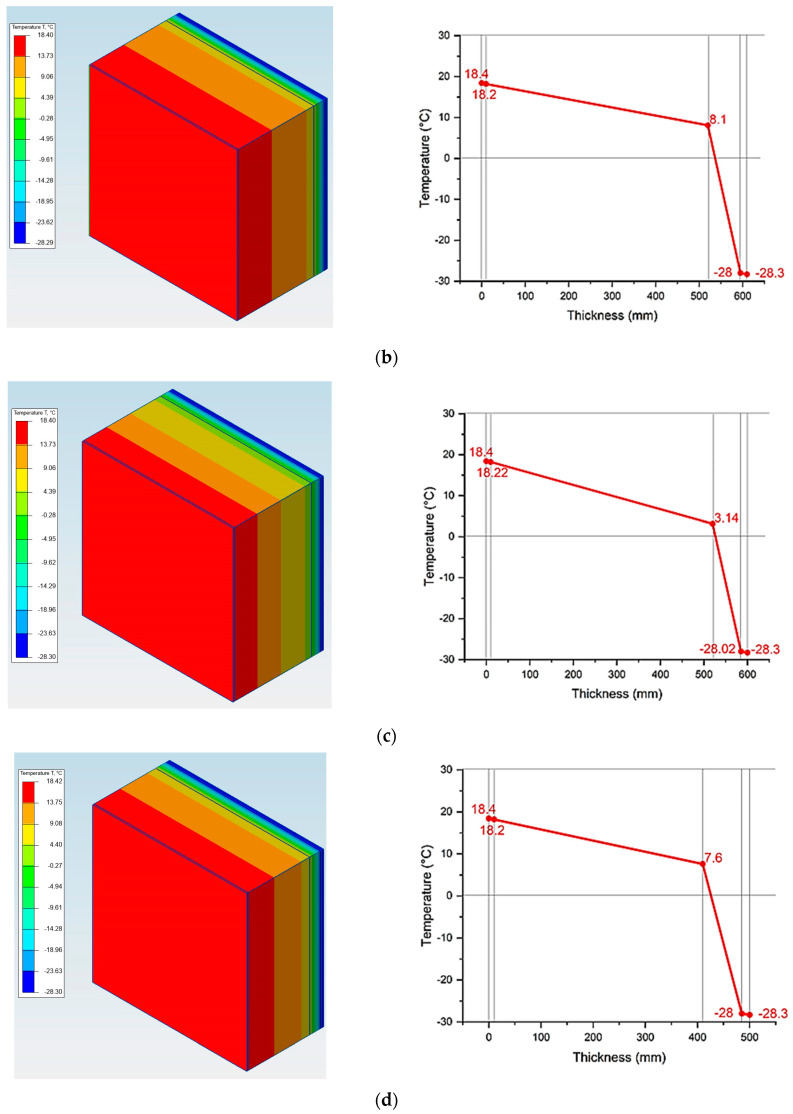

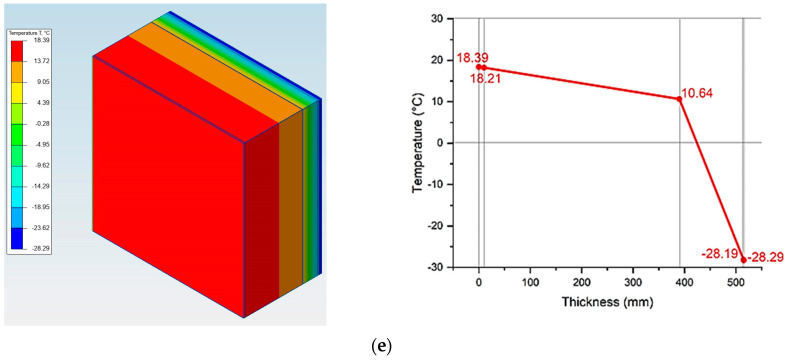
Values of temperature fields of the multilayer structures of the outer shells: (**a**)—three-layer panel made of highly porous polystyrene concrete (option 1); (**b**)—traditional enclosure made of solid ceramic brick (option 2); (**c**)—traditional enclosure made of hollow ceramic brick (option 3); (**d**)—traditional enclosure made of foam block (option 4); (**e**)—outer shell with a ventilated layer (option 5).

Figure 6 shows an analysis of temperature fields of the outer shells, modeled in the ELCUT 6.6 software package [32,33,34,35,36].

### 3.3. Calculation of Humidity Conditions of the Multilayer Structures of the Outer Shells

#### 3.3.1. Calculation of Humidity Condensation in the Multilayer Structures of the Outer Shells

**Figure 7:**
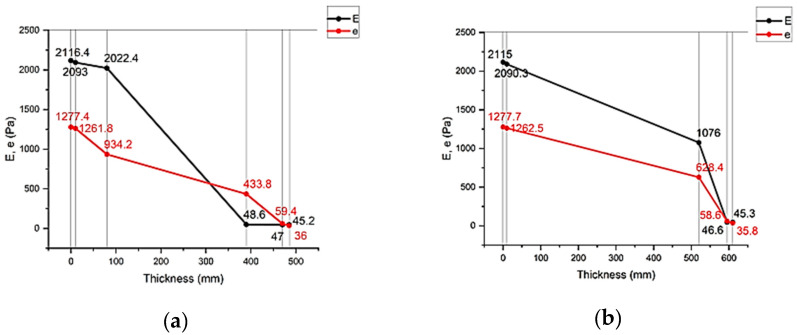

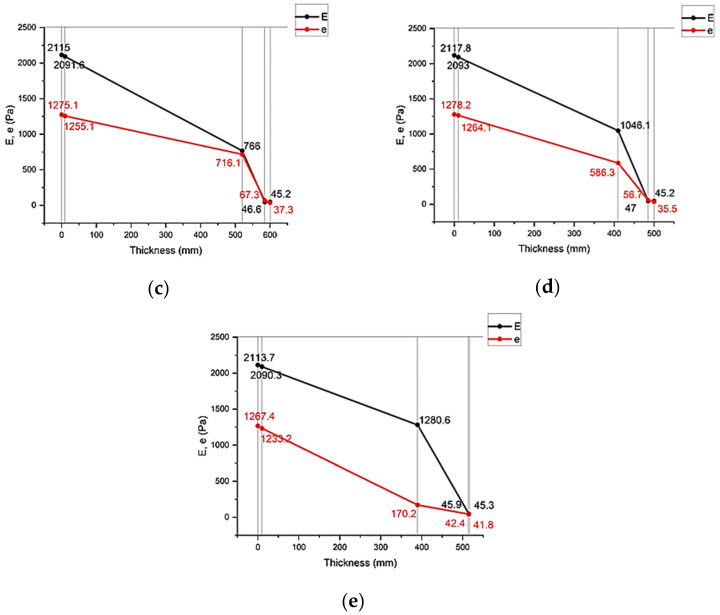
Values of humidity condensation of the multilayer structures of the outer shells: (**a**)—three-layer panel made of highly porous polystyrene concrete (option 1); (**b**)—traditional enclosure made of solid ceramic brick (option 2); (**c**)—traditional enclosure made of hollow ceramic brick (option 3); (**d**)—traditional enclosure made of foam block (option 4); (**e**)—outer shell with a ventilated layer (option 5).

Figure 7 shows the results of the calculation of humidity condensation in the multilayer structures of the outer shells.

#### 3.3.2. Calculation of the Amount of Moisture Condensing in the Multilayer Structures of the Outer Shells During the Period of Moisture Accumulation

**Figure 8:**
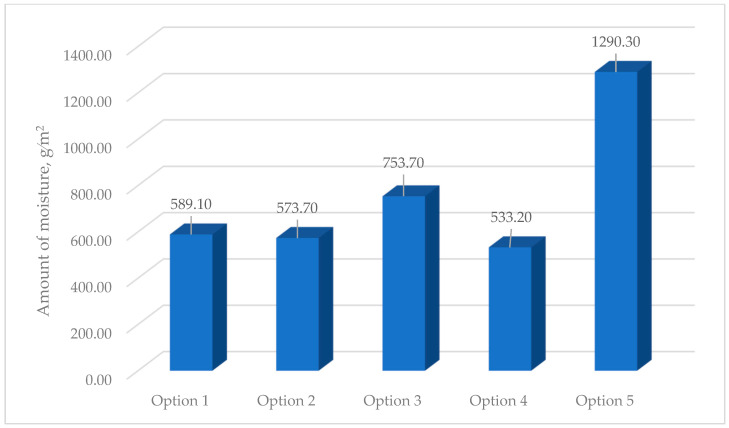
Values of the amount of moisture condensing in the multilayer structures of the outer shells during the period of moisture accumulation: option 1—three-layer panel made of highly porous polystyrene concrete; option 2—traditional enclosure made of solid ceramic brick; option 3—traditional enclosure made of hollow ceramic brick; option 4—traditional enclosure made of foam block; option 5—outer shell with a ventilated layer.

Figure 8 shows the results of calculating the values of the amount of moisture condensing in the multilayer structures of the outer shells during the period of moisture accumulation.

#### 3.3.3. Calculation of the Amount of Moisture Evaporated from the Multilayer Structures of the Outer Shells During the Drying Period

**Figure 9:**
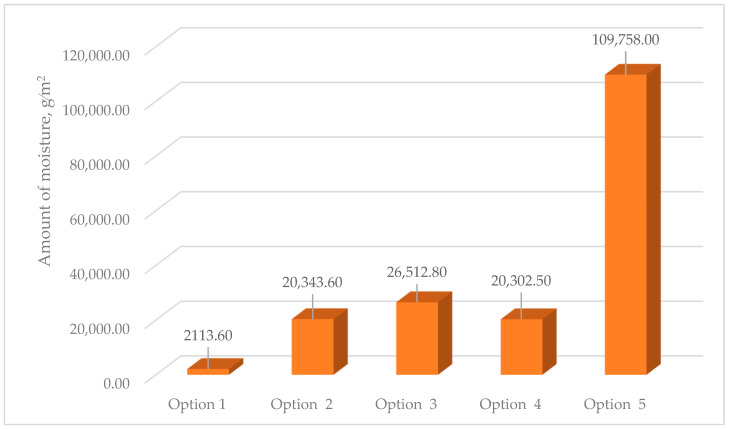
Values of the amount of moisture evaporated from the multilayer structures of the outer shells during the drying period: option 1—three-layer panel made of highly porous polystyrene concrete; option 2—traditional enclosure made of solid ceramic brick; option 3—traditional enclosure made of hollow ceramic brick; option 4—traditional enclosure made of foam block; option 5—outer shell with a ventilated layer.

Figure 9 shows the results of calculating the amount of moisture evaporated from the multilayer structures of the outer shells during the drying period.

#### 3.3.4. Conditions for the Inadmissibility of Moisture Accumulation in the Structures of the Outer Shells over the Annual Period of Operation ($R_{vpc}^{f}\geq R_{vpc}^{req}$)

**Figure 10:**
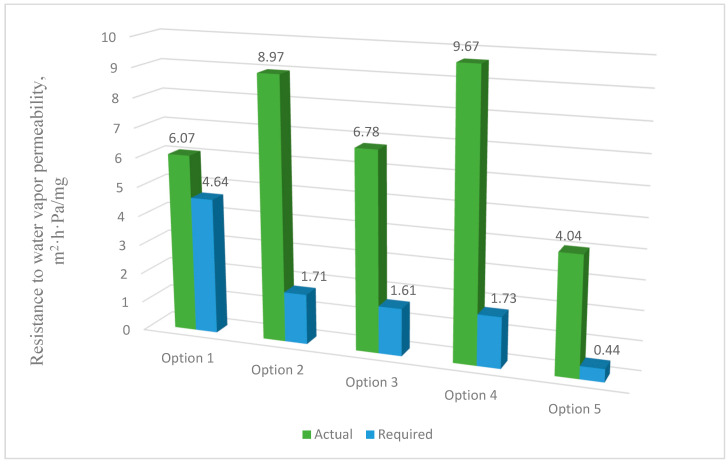
Values of the inadmissibility of moisture accumulation in the multilayer structures of the outer shells over an annual period of operation: option 1—three-layer panel made of highly porous polystyrene concrete; option 2—traditional enclosure made of solid ceramic brick; option 3—traditional enclosure made of hollow ceramic brick; option 4—traditional enclosure made of foam block; option 5—outer shell with a ventilated layer.

Figure 10 shows the calculation of the values of the inadmissibility of moisture accumulation in the multilayer structures of the outer shells over an annual period of operation.

#### 3.3.5. Conditions for the Inadmissibility of Moisture Accumulation in the Multilayer Structures of the Outer Shells During the Period of Moisture Accumulation ($R_{vpc}^{f}\geq R_{vpc}^{req}$)

**Figure 11:**
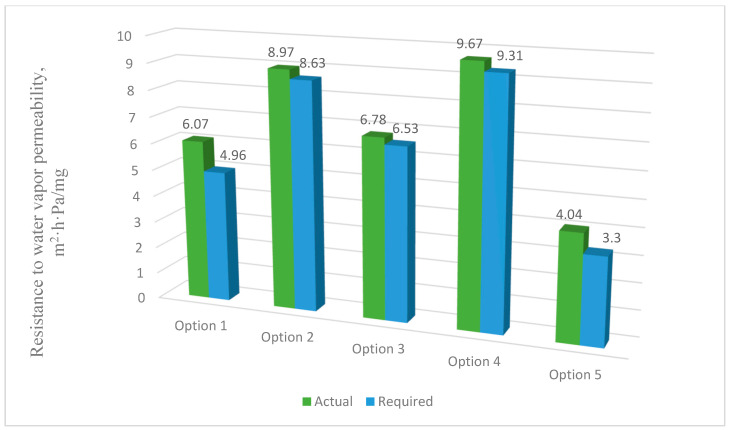
Values of the inadmissibility of moisture accumulation in the multilayer structures of the outer shells during the period of moisture accumulation: option 1—three-layer panel made of highly porous polystyrene concrete; option 2—traditional enclosure made of solid ceramic brick; option 3—traditional enclosure made of hollow ceramic brick; option 4—traditional enclosure made of foam block; option 5—outer shell with a ventilated layer.

Figure 11 shows the results of calculating the values of inadmissibility of moisture accumulation in the multilayer structures of the outer shells during the period of moisture accumulation.

### 3.4. Calculation of Air Conditions in the Multilayer Structures of the Outer Shells ($R_{u}^{req}\leq R_{u}^{f}$)

#### 3.4.1. Calculation of Air Permeability Resistance of the Multilayer Structures of the Outer Shells

Table 7 presents the results of calculating the required and actual air permeability resistances of the multilayer structures of the outer shells.

#### 3.4.2. Research of Temperature Distribution at the Boundaries of the Multilayer Structures of the Outer Shells Taking into Account Air Filtration

Table 8 presents the results of calculating the temperature distributions at the boundaries of the multilayer structures of the outer shells.

### 3.5. Research of Thermal Inertia of the Multilayer Structures of the Outer Shells

**Figure 12:**
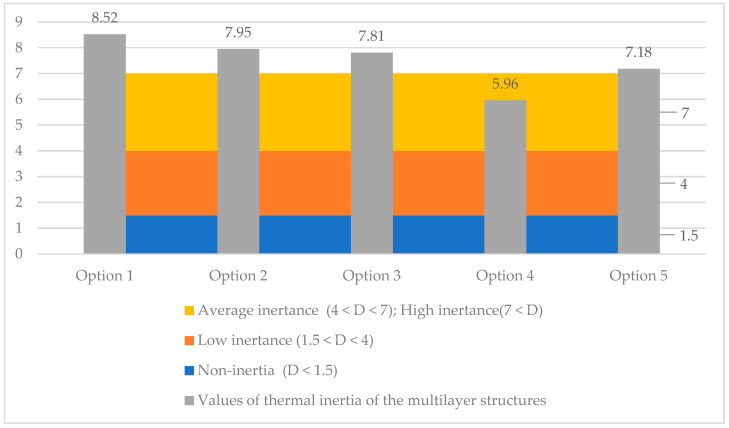
Values of thermal inertia of the multilayer structures of the outer shells: option 1—three-layer panel made of highly porous polystyrene concrete; option 2—traditional enclosure made of solid ceramic brick; option 3—traditional enclosure made of hollow ceramic brick; option 4—traditional enclosure made of foam block; option 5—outer shell with a ventilated layer.

Figure 12 shows the results of calculating the values of thermal inertia of the multilayer structures of the outer shells.

### 3.6. The Market Value of the Construction of the Studied Multilayer Enclosing Structures

**Figure 13:**
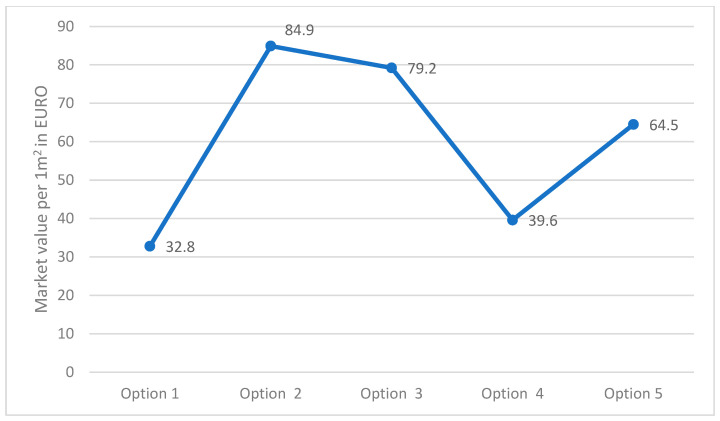
Market values of multilayer structures of the outer shells per 1 m^2^.

Figure 13 shows the market values of the construction of the studied multilayer structures of external fences in the city of Karaganda per 1 m^2^.

This paper presents linking computational research of the multilayer structure of the cladding developed using a new technology—a three-layer panel made of highly porous polystyrene concrete (Figure 1a) [25]—in comparison with traditional ones (Figure 1b–e) using the ELCUT 6.6 software package [32] and the Maple computer algebra system. A multivariate analysis of all the multilayer structures of the outer shells was carried out in three stages, where the first stage analyzed the values of the thermal conditions (Figure 2), and the second and third stages analyzed the values of the humidity (Figure 3) and air (Figure 4) conditions. In the research, the main geometric and thermal engineering characteristics are presented in Figure 1 and Table 1, Table 2, Table 3, Table 4 and Table 5 [27,28]. The central part of the Republic of Kazakhstan (Karaganda) was chosen as the research region, the main climatic values of which were adopted according to [33,34].

The analysis of the first stage of the research showed (Figure 2) that the required heat transfer resistance (R_2_) of the specified region is 3.2 W/$m^{2}\cdot {}^{\circ}$C, adopted according to the value of the degree-day of the heating period according to the standard [27,28]. Taking into account the value of the required heat transfer resistance (R_2_) in the research, the thickness of the multilayer structures of the outer shells was adopted taking into account R_2_, where the values of the actual resistances (R_1_) of heat transfer are presented in Figure 5, which vary in the range from 3.5 to 3.55 W/$m^{2}\cdot {}^{\circ}$C; these values are almost equal at the specified thicknesses of the multilayer structures. Taking these circumstances into account, with the specified (Figure 5) equal accepted values, the thicknesses of traditional enclosures (Figure 1b–e) relative to the developed one (Figure 1a) increase by 25.77%, 23.71%, 3.09%, and 27.83%, respectively. Thus, due to the equating of the enclosure thicknesses, the analysis of temperature fields showed that the temperature on the inner surface of the enclosure is almost all similar, which is equal to 18.40 °C on average (Figure 6).

The analysis of the second stage of the research showed (Figure 3) that moisture accumulation occurs in all structures (Figure 7). With that, the calculation of the amount of accumulated moisture (Figure 8) showed that the values in traditional enclosure options 2 and 4 are 2.61% and 9.48% less, respectively, and for options 3 and 5 they are 27.94% and 119% more, respectively. However, the calculation of the amount of moisture evaporated in the multilayer structures of the outer shells during the drying period showed that all the accumulated moisture in the structures will evaporate (Figure 9). Thus, the condition for the inadmissibility of moisture accumulation in the multilayer structures of the outer shells was additionally analyzed during the annual period of operation (Figure 10) and during the period of moisture accumulation (Figure 11). In all cases, the condition ($R_{vpc}^{1}\geq R_{vpc}^{2}$) is fulfilled, which emphasizes the positive aspect of the developed structure despite the significant difference in thickness relative to traditional ones.

The analysis of the third stage of the research showed (Figure 4) that in all the studied multilayer structures of the outer shells, the value of the required and actual resistance to air permeability satisfies the condition ($R_{u}^{2}\leq R_{u}^{1}$), presented in Table 7. Thus, in the developed structure (Figure 1a) this value is significantly higher than in traditional ones, which had a positive effect on the values of the temperature fields of the enclosure. Thus, in the developed structure (Figure 1a), the temperature of the inner surface ($\tau_{int}$) remains high, even taking into account filtration (18.41 °C); in traditional structures (Figure 1b–e), the value of $\tau_{int}$ decreases to 1.35 °C depending on the option of the traditional multilayer structure, which is an unfavorable factor in the cold period. Moreover, the analytical results of thermal inertia (D) of the developed (Figure 1a) and traditional multilayer structures (Figure 1b–e) showed that the developed structure, according to the value of D, belongs to the high inertia (7 < D) type and exceeds the traditional ones by 6.69%, 8.33%, 30.04% and 15.72%, respectively, which is also a positive moment in the cold period. In conclusion, an analysis of the market value of the studied multilayer structures of external fences was carried out (Figure 13), where it was found that the market prices of all traditional structures (Figure 1b–e) exceed the developed one by 1.2–2.5 times, depending on the design, which also emphasizes the positive aspects of the new design (Figure 1a).

The conducted research on the theoretical study of the developed design of the external fence is part of the research conducted by the authors of [25,31]. As noted above, the efficiency in terms of economic indicators is significant, which is due to the minimum consumption of materials [37,38,39] and rapid construction [40,41]. Since ready-made three-layer panels during installation do not imply a long construction period, as with the structures of ventilated facades [12,13,14,15] and facades made of block masonry [42,43]. In this regard, this type of construction is, by all criteria, the most optimal in the climatic conditions of Karaganda. As a disadvantage of this research, it can be noted that we did not take into account cold joints; however, we will solve and supplement this problem in subsequent research. At the same time, the results obtained in this research will positively complement the catalog of products for multilayer outer shells and can be used by research communities and design organizations in the design of residential buildings.

## 4. Conclusions

This paper presented computational research of a multilayer structure of the outer fence consisting of a three-layer panel made of highly porous polystyrene concrete developed using a new technology in comparison with traditional structures. The study covered such areas as thermal engineering indicators and the economic efficiency of the new design. As a result of a comprehensive study, the following was found:

- Equating the thickness by the R_1_ value taking into account R_2_ of traditional multilayer structures to the developed one, the thickness of traditional structures increases from 3.09% to 27.83% depending on the option, which is ineffective from the point of view of the construction estimate.
- Moisture accumulation relative to the developed structure occurs in all the studied structures in the range of 2113.6–109,758 g/m^2^; if, in options 2 and 4 of traditional structures, moisture is collected by 2.61% and 9.48% less, respectively, then in options 3 and 5 moisture is collected by 27.94% and 119% more, respectively. However, the value of evaporated moisture during the drying period showed that all moisture will evaporate. At the same time, all structures meet the conditions for the inadmissibility of moisture for an annual period and the period of moisture accumulation.
- The values of the actual and required air permeabilities satisfy the condition ($R_{u}^{2}\leq R_{u}^{1}$), which affected the values of the temperature fields taking into account air filtration, where the developed structure showed a positive effect for this value, and in traditional structures the value of $\tau_{int}$ decreased to 1.35 °C depending on the option; this will have an adverse effect in the cold period. The analytical results of the value of D of the developed and traditional multilayer structures showed that the developed structure by its value of D refers to high inertia (7 < D) [25,26] and exceeds traditional ones by up to 30.04% depending on the option, which affected the value of $\tau_{int}$ taking into account filtration. It was also found that the market prices of all traditional structures exceed the developed one by 1.2–2.5 times, depending on the design, which also emphasizes the positive aspects of the new design.

## Figures and Tables

**Table 1:** Thermal engineering characteristics of a three-layer panel made of highly porous polystyrene concrete (option 1), shown in Figure 1a.

№	Layer	Layer Thickness, mm	Thermal Conductivity for Operating Zone A, λ (W/(m·°C)	Heat Absorption, S (W/(m·°C)	Vapor Permeability, μ (mg/m·h·Pa)	Air Permeability Resistance, R_U_ (m^2^·h·Pa/kg)
1	Cement and sand grout	10	0.76	9.6	0.09	373
2	Heavy concrete	70	1.74	16.77	0.03	19,620
3	Polystyrene concrete	310	0.095	2.07	0.087	79
4	Heavy concrete	80	1.74	16.77	0.03	19,620
5	Cement and sand grout	15	0.76	9.6	0.09	373

**Table 2:** Thermal engineering characteristics of the multilayer structure of the outer shell made of solid ceramic brick (option 2), shown in Figure 1b.

№	Layer	Layer Thickness, mm	Thermal Conductivity for Operating Zone A, λ (W/(m·°C)	Heat Absorption, S (W/(m·°C)	Vapor Permeability, μ (mg/m·h·Pa)	Air Permeability Resistance, R_U_ (m^2^·h·Pa/kg)
1	Cement and sand grout	10	0.76	9.6	0.09	373
2	Solid ceramic brick with 1800 kg/m^3^ density	210	0.7	9.2	0.11	18
3	Extruded polystyrene foam with 35 kg/m^3^ density	75	0.029	0.36	0.018	79
4	Cement and sand grout	15	0.76	9.6	0.09	373

**Table 3:** Thermal engineering characteristics of the multilayer structure of the outer shell made of hollow ceramic brick (option 3), shown in Figure 1c.

№	Layer	Layer Thickness, mm	Thermal Conductivity for Operating Zone A, λ (W/(m·°C)	Heat Absorption, S (W/(m·°C)	Vapor Permeability, μ (mg/m·h·Pa)	Air Permeability Resistance, R_U_ (m^2^·h·Pa/kg)
1	Cement and sand grout	10	0.76	9.6	0.09	373
2	Hollow ceramic brick with 1000 kg/m^3^ density	510	0.47	6.16	0.17	2
3	Extruded polystyrene foam with 35 kg/m^3^ density	65	0.029	0.36	0.018	79
4	Cement and sand grout	15	0.76	9.6	0.09	373

**Table 4:** Thermal engineering characteristics of the multilayer structure of the outer shell made of foam block (option 4), shown in Figure 1d.

№	Layer	Layer Thickness, mm	Thermal Conductivity for Operating Zone A, λ (W/(m·°C)	Heat Absorption, S (W/(m·°C)	Vapor Permeability, μ (mg/m·h·Pa)	Air Permeability Resistance, R_U_ (m^2^·h·Pa/kg)
1	Cement and sand grout	10	0.76	9.6	0.09	373
2	Foam block with 1200 kg/m^3^ density	400	0.52	8.17	0.075	196
3	Extruded polystyrene foam with 35 kg/m^3^ density	75	0.029	0.36	0.018	79
4	Cement and sand grout	15	0.76	9.6	0.09	373

**Table 5:** Thermal engineering characteristics of the multilayer structure of the outer shell with a ventilated layer (option 5), shown in Figure 1e.

№	Layer	Layer Thickness, mm	Thermal Conductivity for Operating Zone A, λ (W/(m·°C)	Heat Absorption, S (W/(m·°C)	Vapor Permeability, μ (mg/m·h·Pa)	Air Permeability Resistance, R_U_ (m^2^·h·Pa/kg)
1	Cement and sand grout	10	0.76	9.6	0.09	373
2	Solid ceramic brick with 1800 kg/m^3^ density	380	0.7	9.2	0.11	18
3	Mineral-cotton slabs	125	0.045	0.74	0.3	1.5
4	Hydro-windproof film	-	0.76	-	0.09	150
5	Ventilated air gap	100	-	-	-	-
6	Facing material (composite panels)	5	-	-	-	-

**Table 6:** Climatic and internal boundary conditions.

№	Indicators	Values
1	Development region	Karaganda, Republic of Kazakhstan
2	Humidity conditions of the room	Normal
3	Humidity zone	Dry
4	Operating conditions of cladding structures	A
5	Absolute max. temperature	40.2 °C.
6	Absolute min. temperature	–42.9 °C
7	Average annual temperature	3.7 °C
8	Average temperature of the coldest 5-day period with a probability of 0.92	–28.9 °C
9	Average max. temperature of the warmest month (July)	26.8 °C.
10	Max. amplitude of daily fluctuations in outdoor air temperature in July	12.9 °C
11	Average monthly outdoor air temperature for July	20.4 °C
12	Average monthly temperature of the coldest month (January)	–13.6 °C
13	Average relative humidity of the coldest month (January)	79%
14	Average annual humidity	65%
15	Maximum of average speeds by rhumbs in January	6.6 м/c
16	Duration of the heating season	207 days
17	Internal temperature in winter	20 °C
18	Internal humidity	55%
19	Required design resistance according to the degree-day of the heating period	3.2 W/m^2^ °C

**Table 7:** Values of the required and actual air permeability resistances of the multilayer structures of the outer shells.

№	Schemes	Required Air Permeability Resistance Depending on the Building Height		Actual Air Permeability Resistance	Fulfillment of the Condition
		H = 3 m	H = 15 m		
1	Option—1	43.79	75.07	30,131.91	Done
2	Option—2	43.79	75.07	719.9	Done
3	Option—3	43.79	75.07	703.9	Done
4	Option—4	43.79	75.07	822.9	Done
5	Option—5	43.79	75.07	419.4	Done

**Table 8:** Values of temperature distributions at the boundaries of the multilayer structures of the outer shells.

Condition	τ	Schemes				
		Option 1	Option 2	Option 3	Option 4	Option 5
Without taking into account air filtration, °C according to Figure 3	$\tau_{int}$	18.41	18.40	18.40	18.42	18.39
	$\tau_{1}$	18.23	18.21	18.22	18.23	18.21
	$\tau_{2}$	17.68	8.05	3.14	7.63	10.62
	$\tau_{3}$	−27.39	−28.02	−28.02	−28.03	−28.19
	$\tau_{4}$	−28.03	-	-	-	-
	$\tau_{ext}$	−28.30	−28.29	−28.30	−28.30	−28.29
Taking into account air filtration, °C	$\tau_{int}$	18.41	18.25	18.25	18.29	18.14
	$\tau_{1}$	18.23	18.05	18.05	18.09	17.93
	$\tau_{2}$	17.67	7.21	2.09	6.87	9.40
	$\tau_{3}$	−27.40	−28.10	−28.10	−28.10	−28.29
	$\tau_{4}$	−28.03	-	-	-	-
	$\tau_{ext}$	−28.30	−28.35	−28.35	−28.35	−28.38
Difference	%	Up to 0.5	10.4	33.4	9.96	11.5

## Data Availability

Data are contained within the article.

## Nomenclature

$R_{1}$	Actual heat transfer resistance of multilayer structures of outer shells, $(m^{2}\cdot {}^{\circ}C$)/W;
$\alpha_{int}$	Heat transfer coefficient of the inner surface of the cladding structure, $\alpha_{int}=8.7\;W/\left(m^{2}\cdot {}^{\circ}C\right)$;
$\alpha_{ext}$	Heat transfer coefficient of the outer surface of the cladding structure, $\alpha_{ext}=23\;W/(m^{2}\cdot {}^{\circ}C);$
$R_{s}$	Thermal resistance of the layer of the fragment’s homogeneous part, $(m^{2}\cdot {}^{\circ}C$)/W;
$\delta_{s}$	layer thickness, m;
$\lambda_{s}$	Thermal conductivity of the layer material under the operating conditions of structure A, $W/\left(m^{2}\cdot {}^{\circ}C\right)$;
$\tau_{x}$	Temperature in any section of the cladding structure, °C;
$R_{x}$	Resistance to heat transfer of the layers of the structure from the internal air to the section under consideration, $(m^{2}\cdot {}^{\circ}C$)/W;
$t_{int}$	Indoor air temperature, °C;
$t_{ext}$	Outside air temperature, °C;
$e_{x}$	Actual elasticity of water vapor in any section of the cladding structure, Pa;
$e_{int}$	Actual elasticity of water vapor in the internal air of the cladding structure, Pa;
$e_{ext}$	Actual elasticity of water vapor in the outside air of the cladding structure, Pa;
$R_{ovp}$	Resistance to vapor permeability of a single layer or separate layer of a multilayer outer shell, $m^{2}\cdot h\cdot \mathrm{Pa}/\mathrm{mg}$;
$R_{xvp}$	Vapor permeability resistance of layers from the inner surface of the wall to section x;
$R_{vp}$	Vapor permeability resistance of a single layer or separate layer of the multilayer cladding structure, $m^{2}\cdot h\cdot \mathrm{Pa}/\mathrm{mg}$;
δ	Layer thickness, m;
μ	Calculated vapor permeability coefficient of the layer material, mg/(m·h·Pa);
$R_{vpint},\;R_{vpext}$	Vapor permeability resistance of the inner and outer wall surfaces, respectively, $m^{2}\cdot h\cdot \mathrm{Pa}/\mathrm{mg}$;
$\varphi_{int},\;\varphi_{ext}$	Relative humidity of the indoor and outdoor air, respectively, %;
$E_{int},\;E_{ext}$	Saturated partial pressure at the temperature of the indoor and outdoor air, respectively, Pa;
$G_{c}$	Amount of moisture condensing in the multilayer structure of the outer shell during the period of moisture accumulation, g/m^2^;
$e_{int}$	Actual elasticity of water vapor in the internal air of the cladding structure, Pa;
$e_{c}$	Actual elasticity of water vapor in the plane of possible condensation of the cladding structure, Pa;
$Z_{con}$	Duration of the condensation period, hours;
$R_{vpc}$	Vapor permeability resistance of the cladding structure part from the inner surface to the condensation plane, $m^{2}\cdot h\cdot \mathrm{Pa}/\mathrm{mg}$;
$\overset{n}{\underset{i=1}{\sum }}R_{i}$	Vapor permeability resistance of the cladding structure part from the inner surface to the condensation plane, $m^{2}\cdot h\cdot \mathrm{Pa}/\mathrm{mg}$;
$G_{u}$	Amount of moisture evaporated from the multilayer structure of the outer shell during the drying period, g/m^2^;
$e_{ext}$	Actual humidity of the outside air during the drying period, Pa;
$E_{c}$	Saturated water vapor pressure at the average temperature of the drying period, Pa;
$Z_{u}$	Duration of the drying period, hours;
$R_{vpcext}$	Resistance to vapor permeability of the part of the cladding structure between the plane of possible condensation and the outer surface of the cladding structure, $m^{2}\cdot h\cdot \mathrm{Pa}/\mathrm{mg}$;
E	Elasticity of water vapor in the plane of possible condensation over an annual period, Pa;
$E_{1},\;E_{2},\;E_{3}$	Saturated pressure of water vapor, according to the temperatures of winter, spring-autumn, summer periods, respectively, Pa;
$Z_{1},\;Z_{2},\;Z_{3}$	Duration of winter, spring, autumn, summer periods, respectively, months;
$\gamma_{\omega }$	Density of the material of the moistened layer, kg/m^3^;
$\delta_{\omega }$	Thickness of the moistened layer, m;
$\Delta W_{\omega }$	Maximum permissible increase in the calculated mass ratio of moisture in the material of the moistened layer, %;
$E_{o}$	Partial pressure of the condensation zone during the period of moisture accumulation, Pa;
$Z_{o}$	Duration of condensation period, months;
$R_{u}^{req}$	Required air permeability resistance,$\;Pa\cdot m^{2}\cdot h/\mathrm{kg};$
$R_{u}^{f}$	Actual air permeability resistance, $Pa\cdot m^{2}\cdot h/\mathrm{kg};$
Δp	Calculated value of the total pressure difference due to temperature difference and wind, Pa;
$G_{H}=0.5$	Transverse air permeability for external walls, $\mathrm{kg}/m^{2}\cdot h;$
$\gamma_{ext},\;\gamma_{int}$	Density of cold and warm air, respectively, kg/m^3^;
H	Building height (from the floor level of the first floor to the top of the exhaust shaft), m;
ϑ	Maximum of average wind speeds by rhumbs for January, m/s;
e	Base of natural logarithms;
G	Amount of air filtered through the structure per unit of time, kg/(m^2^·h);
c =1	Specific heat capacity of air;
D	Thermal inertia;
s	Heat absorption, W/$(m^{2}\cdot {}^{\circ}C$).
